# North London Hospital for Consumption

**Published:** 1894-06-09

**Authors:** 


### NORTH LONDON HOSPITAL FOR CONSUMPTION.

The friends and supporters of this hospital to the number of some one hundred and twenty gathered at the Hôtel Métropole on Saturday night under the presidency of Mr. Alfred Hoare, L.C.C., the treasurer. Among those present were—most of them being active workers on behalf of the institution—the Rev. Arthur Godson, Sir J. Kennaway, M.P., Mr. T. Shepherd Little, M.P., Captain Battiscombe, Mr. A. Lyon, chairman of the Board, Captain R. Ellis, Mr. J. S. Fletcher, L.C.C., Mr. J. V. Fitzgerald, Mr. Forbes Robertson, Dr. Forbes Winslow, Dr. Douglas Pidcock, Dr. Bernard O'Connor, Dr. Gerald, Dr. J. Edward Squire, Dr. Fletcher Little, Dr. Fred Weaver, Dr. F. R. Watvers, Dr. J. G. Dudley, Mr. Alfred Essex, Mr. W. F. Malcolm, and Mr. Chas. Johnston. The Hampstead hospital is virtually in the position of having to face sooner or later, probably sooner, a very urgent appeal to the public for means to remove a heavy mortgage of £8,000 now resting upon the buildings. The actual yearly income

Hospital Festivals and Meetings—*continued*.

tells much the same tale as the figures of most similar institutions can tell and are telling at the present time, an annual subscription list normal and ever improving despite the condition of trade, a dwindling total of annual donations, and a falling off in legacies, this particular diminution being actually from £3,938 in 1892 to *nil* in 1893. Still, taken all round, the results are encouraging, and provided the mortgage could be, at least to some extent, wiped out, and the new central block, which will accommodate 35 extra patients—there were 2,894 out and 378 in patients treated last year—the institution would have a better chance of going ahead and making the progress which must assuredly in the future attend its labours. It was a good sign that the chairman's cheery, optimistic speech had a thoroughly warm reception. This, indeed, was practically the sole oration of any noteworthiness. Royalty was disposed of in one omnibus toast, and then the Chairman, in proposing "Success and Prosperity to the Hospital," pointed out that their institution was one of which they had every reason to be proud, not only for its usefulness, but even as a monument of architecture. (Cheers.) Their income for 1893 was only £3,375, including subscriptions and donations, whilst their expenses were £5,411, leaving a deficit in the shape of debt of £2,036, besides the additional mortgage debt on the hospital. Moreover, the committee had been unfortunate in not coming in for any legacies in the past year. The North London Hospital had no endowments whatever. It was founded in 1860, and it had been gradually extended and developed until two years before the central block was added. For this block the furniture had yet to be found, and additional income provided to keep the hospital in an efficient state, and when this was done they hoped that their work might be extended further. After observing that if properly approached some of the neighbouring authorities might perhaps be induced to subscribe, Mr. Hoare proceeded to show that the addition of the central block would mean an increase in the accommodation from 84 to some 120 beds, and while he did not wish to say anything to deter anyone who was endeavouring to support the Brompton and City Road institutions, he thought the benevolence of the public would be better employed for the development of the work at Hampstead than it could be in any other direction. A peculiarity observable in the work of their hospital in a more marked degree than in other institutions was the success with which the medical profession during the last 20 or 30 years had been conducting the battle against disease, and they stated now that they were more certain than ever before that phthisis could be combatted successfully, one important factor in the struggle being the effect of light and air. Those connected with that hospital felt they were carrying on a successful battle, and it was particularly on that ground that they felt themselves the more justified in appealing more strongly than ever before for the financial assistance which was requisite for their support. (Cheers.)

The toasts having been drunk with great heartiness, Mr. J. S. Fletcher proposed "The Health of the Chairman," pointing out incidentally that there was a serious difficulty in the way of the neighbouring authorities contributing as Mr. Hoare had suggested they should to an institution which did not confine its labours to the Hampstead people. He did, however, think the time was approaching when the London hospitals would need some assistance from the rates of the metropolis, and when it did come, he considered that it would be the duty of the London County Council to take the matter into their consideration. He sometimes thought that they might proceed on the just principle of assisting the institutions *pro rata* to the amount collected by voluntary subscriptions, whether it was a fifth, a third, or a half. The toast was drunk with musical honours, but it did not, as usual, close the speechmaking, for, after the secretary had announced contributions to the amount of £1,243, the Rev. A. Godson proposed "The Health of the Managing Committee and the Stewards." The replies of Mr. B. A. Lyon and Mr. T. S. Little, M.P., were commendably short. The former gentleman, however, gave, in a cheery tone, a rather gloomy picture of the difficulty experienced in collecting funds, and then a well arranged musical programme brought the proceed ings to a close.

